# Risk ranking of foodborne parasites: State of the art

**DOI:** 10.1016/j.fawpar.2017.11.001

**Published:** 2017-11-23

**Authors:** Brecht Devleesschauwer, Martijn Bouwknegt, Pierre Dorny, Sarah Gabriël, Arie H. Havelaar, Sophie Quoilin, Lucy J. Robertson, Niko Speybroeck, Paul R. Torgerson, Joke W.B. van der Giessen, Chiara Trevisan

**Affiliations:** a Department of Public Health and Surveillance, Scientific Institute of Public Health (WIV-ISP), Brussels, Belgium; b Group Quality Assurance/R&D, Vion, Boxtel, The Netherlands; c Department of Biomedical Sciences, Institute of Tropical Medicine, Antwerp, Belgium; d Laboratory of Parasitology, Faculty of Veterinary Medicine, Ghent University, Merelbeke, Belgium; e Department of Veterinary Public Health and Food Safety, Faculty of Veterinary Medicine, Ghent University, Merelbeke, Belgium; f Emerging Pathogens Institute, Institute for Sustainable Food Systems, Animal Sciences Department, University of Florida, Gainesville, FL, USA; g Parasitology, Department of Food Safety and Infection Biology, Faculty of Veterinary Medicine, Norwegian University of Life Sciences, Adamstuen Campus, Oslo, Norway; h Institute of Health and Society (IRSS), Université Catholique de Louvain, Brussels, Belgium; i Section of Epidemiology, Vetsuisse Faculty, University of Zürich, Switzerland; j Centre for Zoonoses and Environmental Microbiology, National Institute for Public Health and the Environment, Bilthoven, The Netherlands

**Keywords:** DALY, Disability-Adjusted Life Year, FAO, Food and Agriculture Organization of the United Nations, GBD, Global Burden of Disease, MCDA, Multi-Criteria Decision Analysis, QALY, Quality-Adjusted Life Year, SMPH, Summary Measure of Population Health, WHO, World Health Organization, WTA, Willingness-to-accept, WTP, Willingness-to-pay, YLD, Year Lived with Disability, YLL, Year of Life Lost, Cost-of-illness, Disability-adjusted life years, Foodborne parasites, Multi-criteria decision analysis, Priority setting

## Abstract

In a time of increasing threats and decreasing financial resources, monitoring and controlling all possible foodborne hazards at the same time and to the same extent has become more challenging than ever. Therefore, attention is increasingly being paid to the so-called “risk ranking” methods that enable decision makers to focus on the most important foodborne hazards — even when time is limited and knowledge incomplete. In this review paper, we provide an overview of the most common quantitative methods and metrics used for ranking the risks associated with foodborne parasites and present the state of the art on risk ranking exercises for foodborne parasites.

A number of risk ranking metrics and methods are available, ranging from simple approaches that can be used to assess the health or economic impact of a foodborne parasitic disease, to more complicated but more comprehensive multi-criteria assessments. For health impact assessment, measures of population health such as disease occurrence and number of deaths; Disability-Adjusted Life Years (DALYs) measuring the healthy life years lost; and Quality-Adjusted Life Years (QALYs) measuring the number of life years lived in optimal health, are described. For economic impact assessment, applied approaches that measure the cost-of-illness from a societal perspective and stated preference methods are outlined. Finally, Multi-Criteria Decision Analysis (MCDA), which can be used to integrate multiple metrics and criteria into a single ranking, is described.

These risk ranking methods for foodborne parasites are increasingly performed to aid priority setting at global, regional, and national levels. As different stakeholders have their own prioritization objectives and beliefs, the outcome of such exercises is necessarily context-dependent. Therefore, when designing a risk ranking exercise for foodborne parasites, it is important to choose the metrics and methods, as well as what to rank, in the light of the predefined context of the question being addressed and the target audience.

## Highlights

• Risk ranking of foodborne parasites is increasingly used to set priorities.
• Methods range from simple metrics to comprehensive multi-criteria assessments.
• The choice of methods and the prior definition of what to rank define the outcomes.
• The design of risk ranking exercises critically depends on the target audience.

## 1 Introduction

In a time of increasing threats (or recognition, c.q., perception thereof) and decreasing financial resources, it has become more challenging than ever to monitor and control all possible foodborne hazards at the same time and to the same extent (Speybroeck et al., 2015). Consequently, attention is being increasingly directed on methods that enable decision makers to focus on the most important foodborne hazards — even when time is limited and knowledge incomplete (Stella et al., 2013). These exercises are often labeled “risk ranking”, but may differ widely in their intention, scope and methodology. According to the Codex Alimentarius, risk is defined as “a function of the probability of an adverse health effect and the severity of that effect, consequential to a hazard(s) in food” (CAC (Codex Alimentarius Commission), 1999). However, severity can be quantified in different ways — it may, for instance, be defined as the health or economic impact of the adverse health effects. Furthermore, the function can take many different shapes — ranging from a mere sum to complicated weighted averages. As a result, the concept of “risk”, and thus “risk ranking”, is not as standardized as it should be. However, the same goal, which is to accomplish an internally consistent and comparable set of risk estimates allowing ranking, and thus prioritization among a given number of hazards, is shared in all risk ranking exercises.

Foodborne parasitic diseases present some unique challenges, including their often prolonged incubation period and association with chronic sequelae. Furthermore, as most foodborne parasitic diseases are not notifiable, their true importance is often underreported and under-recognized (Torgerson et al., 2015). In this review paper, we aim to provide an overview of the most common quantitative methods and metrics used for ranking foodborne parasites according to their associated risks. We also provide the state of the art on risk ranking exercises for foodborne parasites. For further information on risk ranking, readers are kindly referred to Brookes et al. (2015), who discuss risk ranking in the context of decision science, and to O'Brien et al. (2016) and Van der Fels-Klerx et al. (2016), who discuss risk ranking methods for infectious and foodborne diseases, respectively.

## 2 Health impact

### 2.1 Methods and metrics

Quantifying health impacts may be based on disease occurrence (prevalence or incidence) or on the number of deaths (mortality). However, these unidimensional or *simple* measures of population health do not provide a complete picture of the impact of foodborne parasites on human health as they do not combine the impacts of morbidity and mortality, thus precluding a comparative ranking of diseases with high morbidity, but low case-fatality, such as chorioretinitis due to toxoplasmosis, and highly lethal diseases such as alveolar echinococcosis (Batz et al., 2012, Devleesschauwer et al., 2015a). Furthermore, disease severity, defined by the impact on quality of life and the duration of the symptoms, as well as the expected residual life expectancy at the age of death, should be accounted for when quantifying burden of disease. Indeed, certain parasitic infections may be very common, but their clinical impact may be minimal. For instance, infections with a highly prevalent parasite such as the pinworm, *Enterobius vermicularis*, have a very low burden because most of the cases are mild to asymptomatic and self-limiting (Knopp et al., 2012).

In order to overcome the limitations of simple measures such as incidence and mortality, *summary* measures of population health (SMPHs) have been developed as an additional way of expressing information for quantifying disease burden. The Disability-Adjusted Life Year (DALY) is currently the most widely used SMPH in public health research. Originally developed to quantify and compare the burden of diseases, injuries, and risk factors within and across countries, the DALY summarizes the occurrence and impact of morbidity and mortality in a single metric (Devleesschauwer et al., 2014a). The DALY is the key measure in the Global Burden of Disease (GBD) studies and has been officially adopted by the World Health Organization (WHO) for reporting on health information (Murray et al., 2012; WHO (World Health Organization), 2017).

The DALY is a health gap measure, measuring the quantity of healthy life years lost due to a disease or injury against some idealized health profile. DALYs are calculated by adding the number of years lived with disability adjusted for the severity of the disease (YLDs) and the number of years of life lost due to premature mortality (YLLs):

*YLD* = *Number of incident cases* × *Duration until remission or death* × *Disability Weight*.

*YLL* = *Number of deaths* × *Residual life expectancy at the age of death*.

An alternative formula for calculating YLDs was introduced by the GBD 2010 study (Murray et al., 2012):

*YLD* = *Number of prevalent cases* × *Disability Weight*.

This formula reflects a prevalence perspective instead of an incidence perspective. The incidence perspective assigns all health outcomes, including those in future years, to the initial event (e.g., exposure to a certain foodborne parasite). This approach therefore reflects the future burden of disease resulting from current events. In the prevalence perspective, on the other hand, the health status of a population is assessed at a specific point in time, and prevalent diseases are attributed to initial events that happened in the past. This approach thus reflects the current burden of disease resulting from previous events. Although both perspectives are valid, the incidence perspective is more appropriate for foodborne parasites, as it is more sensitive to current epidemiological trends, including the effects of intervention measures (Murray, 1994, Devleesschauwer et al., 2015a).

**Fig. 1:**
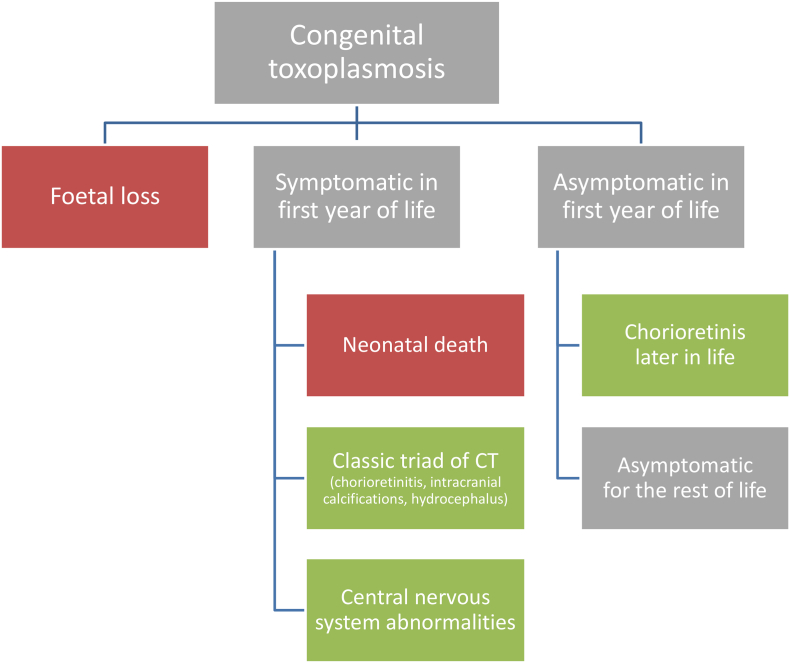
Disease model for congenital toxoplasmosis (CT), adapted from Nissen et al. (2017). Green boxes accrue years lived with disability, red boxes accrue years of life lost. (For interpretation of the references to colour in this figure legend, the reader is referred to the web version of this article.)

Different approaches can be used for calculating DALYs, depending on whether the interest lies in quantifying the burden of a health outcome, hazard, or risk factor (Devleesschauwer et al., 2014b). An obvious choice for quantifying the health impact of foodborne parasites is the *hazard*-*based* approach. This approach defines the burden of a specific foodborne parasites as that resulting from the health states, i.e., acute symptoms, chronic sequelae, and death, which are causally related to the concerned parasite transmitted through food, and which may become manifest at different time scales or have different severity levels (Mangen et al., 2013). The starting point for quantifying DALYs is therefore typically the construction of a *disease model* or outcome tree, which is a schematic representation of the various health states associated with the concerned hazard, and the possible transitions between these states (Devleesschauwer et al., 2014b). Fig. 1 presents an example disease model for congenital toxoplasmosis, but excludes the potential for long-term psychiatric outcomes or the potential for reactivation of infection in people who develop immunosuppressive disorders.

The key epidemiological data required by DALYs are the number of cases for all considered health states, including death, which are associated with the foodborne parasite under study. This requires that an association has to be established, and quantified, between the health state and the foodborne parasite. Two complementary approaches may be defined for quantifying foodborne parasite attributable cases (Stella et al., 2013, Devleesschauwer et al., 2015a). The top-down approach starts from available epidemiological data, and associates health states with the concerned hazard at an individual level (i.e., categorical attribution) or at a population level (i.e., comparative risk assessment based on the calculation of population attributable fractions). The bottom-up approach starts from exposure and dose-response data, and predicts the expected number of cases through risk assessment (see, for instance, Anantanawat et al., 2012). Although both methods build on solid methodological foundations, they have been shown to provide differing estimates for chemical (Jakobsen et al., 2015) and microbial hazards (Evers and Bouwknegt, 2016), and there are no indications that this would not be the case for foodborne parasites.

In addition to the DALY metric, other SMPHs may be used to quantify the health impact of foodborne parasites, such as the Quality-Adjusted Life Year (QALY) metric. QALYs are a measure of the number of life years lived in optimal health, obtained by integrating quantity and quality of life:

*QALY* = *Duration* × *Health*-*related quality of life weight*.

Although the QALY is a key metric in cost-effectiveness analyses (Sanders et al., 2016), its use in burden of disease assessments has so far remained limited. Batz et al. (2012), for instance, compared QALYs in presence versus in absence of a certain foodborne parasitic illness, thereby obtaining an estimate of the QALY losses due to the foodborne parasite, and thus a measure of health impact.

Although SMPHs provide a clear advantage over unidimensional measures of population health, they come at a cost of being more data-demanding, making them more prone to biases and uncertainties. Furthermore, the integration of morbidity and mortality necessitates normative assumptions, for instance regarding death being the worst health state to experience. Such assumptions result in methodological differences for calculating a given SMPH, as well as sparking continuous debate among experts (Gold et al., 2002). Moreover, SMPHs per definition only quantify tangible health impact, thus ignoring economic impact and perceived burden. Finally, the aggregated nature of SMPHs also makes it difficult to understand which factors drive a high burden (incidence, severity, mortality, age) and estimates should always be accompanied by disaggregated data.

### 2.2 State of the art

**Fig. 2:**
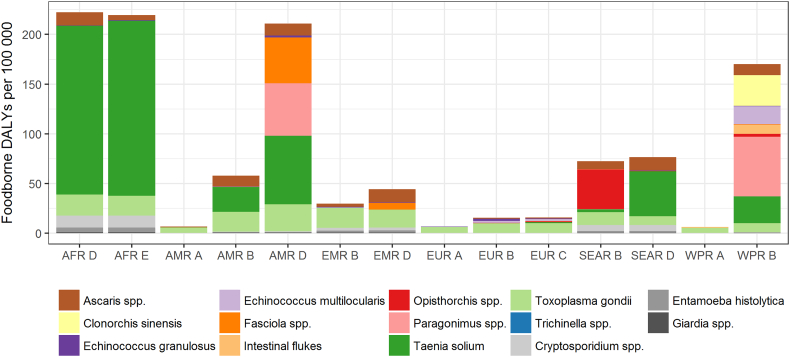
Foodborne Disability-Adjusted Life Years (DALYs) per 100,000 population, per World Health Organization sub-region (Torgerson et al., 2015, Kirk et al., 2015). AFR: African Region, AMR: Region of the Americas, EMR: Eastern Mediterranean Region, EUR: European Region, SEAR: South-East Asia Region, WPR; Western Pacific Region; Stratum A: very low child and adult mortality, Stratum B: low child mortality and very low adult mortality, Stratum C: low child mortality and high adult mortality, Stratum D: high child and adult mortality, Stratum E: high child mortality and very high adult mortality.

To date, the most comprehensive risk ranking of foodborne parasites based on health impact has been achieved by the Foodborne Disease Burden Epidemiology Reference Group (FERG) of the World Health Organization. FERG quantified the global and regional burden of 31 foodborne hazards (Havelaar et al., 2015), including four protozoa (*Cryptosporidium* spp., *Entamoeba histolytica*, *Giardia* spp., and *Toxoplasma gondii*) and ten helminths (including two nematodes: *Ascaris* spp., *Trichinella* spp.; three cestodes: *Echinococcus granulosus*, *Echinococcus multilocularis*, *Taenia solium*; and five trematodes: *Clonorchis sinensis*, *Fasciola* spp., intestinal flukes, *Opisthorchis* spp., *Paragonimus* spp.) (Kirk et al., 2015, Torgerson et al., 2015). Data were abstracted from systematic reviews, disease databases, and reports from national surveillance systems, and used to estimate the number of infections, sequelae, deaths, and DALYs, by age and region for 2010. A Bayesian random effects model was used to impute data gaps, while expert elicitation was used to attribute disease burden to different exposure routes and food items. Together, the considered parasitic diseases caused more than 400 million illnesses, resulting in nearly 100,000 deaths and 12 million DALYs. Intestinal protozoa were responsible for nearly 90% of illnesses, while helminths were responsible for the majority (60%) of deaths and DALYs. Across all parasites being considered, 22% of illnesses, 55% of deaths, and 61% of DALYs were estimated to be due to foodborne transmission. The highest numbers of global foodborne deaths were due to *T*. *solium*, *E*. *multilocularis*, and *C*. *sinensis*; while the highest numbers of global foodborne DALYs were due to *T*. *solium*, *Paragonimus* spp., and *T*. *gondii* (Table 1). The largest burden of foodborne parasitic disease occurred in the African sub-regions and the developing sub-regions of the Americas and Southeast Asia (Fig. 2).

**Table 1:** World Health Organization Foodborne Disease Burden Epidemiology Reference Group (WHO/FERG) estimates of the global burden of fourteen foodborne parasitic hazards, 2010 (Torgerson et al., 2015, Kirk et al., 2015).

Cases (‘000)	Deaths	Disability-adjusted life years (‘000)
*Giardia* spp. (28236)	*Taenia solium* (28114)	*Taenia solium* (2788)
*Entamoeba histolytica* (28024)	*Echinococcus multilocularis* (7771)	*Paragonimus* spp. (1049)
*Toxoplasma gondii* (10280)	*Clonorchis sinensis* (5770)	*Toxoplasma gondii* (829)
*Ascaris* spp. (12281)	*Cryptosporidium* spp. (3759)	*Ascaris* spp. (605)
*Cryptosporidium* spp. (8585)	*Opisthorchis* spp. (1498)	*Clonorchis sinensis* (523)
*Taenia solium* (370)	*Entamoeba histolytica* (1470)	*Echinococcus multilocularis* (312)
*Paragonimus* spp. (139)	*Ascaris* spp. (1008)	*Cryptosporidium* spp. (296)
*Echinococcus granulosus* (43)	*Toxoplasma gondii* (684)	*Opisthorchis* spp. (188)
*Clonorchis sinensis* (32)	*Echinococcus granulosus* (482)	Intestinal flukes (155)
Intestinal flukes (19)	*Paragonimus* spp. (250)	*Entamoeba histolytica* (139)
*Opisthorchis* spp. (16)	*Trichinella* spp. (4)	*Fasciola* spp. (90)
*Fasciola* spp. (11)	*Fasciola* spp. (0)	*Echinococcus granulosus* (40)
*Echinococcus multilocularis* (8)	*Giardia* spp. (0)	*Giardia* spp. (26)
*Trichinella* spp. (4)	Intestinal flukes (0)	*Trichinella* spp. (0.6)

In addition to the FERG estimates, internally comparable estimates of the global, regional and national health burden of foodborne parasites have been generated by the Institute for Health Metrics and Evaluation (GBD 2016 DALYs and HALE Collaborators, 2017). In the Global Burden of Disease 2016 study, five foodborne parasitic diseases were included – i.e., cysticercosis, cystic echinococcosis, foodborne trematodoses, cryptosporidiosis, and amoebosis. Table 2 shows the estimated number of YLDs, deaths and DALYs for the year 2016.

**Table 2:** Institute for Health Metrics and Evaluation (IHME) estimates of the global burden of five foodborne parasitic diseases (GBD 2016 DALYs and HALE Collaborators, 2017; IHME (Institute for Health Metrics and Evaluation), 2016).

Years lived with disability (‘000)	Deaths	Disability-adjusted life years (‘000)
Foodborne trematodoses (1771)	Cryptosporidiosis (57203)	Cryptosporidiosis (4610)
Cysticercosis (421)	Amoebosis (26748)	Foodborne trematodoses (1771)
Amoebosis (207)	Cystic echinococcosis (1012)	Amoebosis (1277)
Cryptosporidiosis (117)	Cysticercosis (999)	Cysticercosis (468)
Cystic echinococcosis (90)	Foodborne trematodoses (0)	Cystic echinococcosis (137)

In addition to global and regional risk ranking exercises, several authors have estimated the burden of foodborne parasites at country level to support national decision making (Haagsma et al., 2013). Table 3 provides an overview of comparative burden of disease studies conducted at national level. These studies were mainly set in developed countries (i.e., the Netherlands, United States, Canada, Greece), while two were set in Nepal and Kyrgyzstan. Most included *T*. *gondii*, *Cryptosporidium* spp., *Giardia* spp., and a few included *E*. *granulosus*, *E*. *multilocularis*, *Entamoeba* spp., and *Cyclospora cayetanensis*.

**Table 3:** National risk ranking of foodborne parasites based on summary measures of population health.

Reference	Country	Reference period	Scope	Foodborne parasite (disease); ranked from highest to lowest estimated burden
Gkogka et al., 2011	Greece	1996–2006	19 foodborne diseases	*Echinococcus granulosus* (cystic echinococcosis) *Toxoplasma gondii* (congenital toxoplasmosis) N/A (“other helminthoses”) *Cryptosporidium* spp. (cryptosporidiosis) *Giardia* spp. (giardiosis) *Entamoeba* spp. (amoebosis)
Hoffmann et al., 2012	United States of America	2009	14 foodborne pathogens	*Toxoplasma gondii* (acquired and congenital toxoplasmosis) *Cryptosporidium* spp. (cryptosporidiosis) *Cyclospora cayetanensis* (cyclosporosis)
Havelaar et al., 2012	The Netherlands	2009	14 foodborne pathogens	*Toxoplasma gondii* (acquired and congenital toxoplasmosis) *Giardia* spp. (giardiosis) *Cryptosporidium* spp. (cryptosporidiosis)
Kwong et al., 2012	Ontario, Canada	2006	51 infectious diseases	*Giardia* spp. (giardiosis) *Cryptosporidium* spp. (cryptosporidiosis) *Cyclospora cayetanensis* (cyclosporosis)
Devleesschauwer et al., 2014c	Nepal	2000–2012	3 parasitic zoonoses	*Taenia solium* (neurocysticercosis) *Toxoplasma gondii* (congenital toxoplasmosis) *Echinococcus granulosus* (cystic echinococcosis)
Mangen et al., 2015	The Netherlands	2011	14 foodborne pathogens	*Toxoplasma gondii* (acquired and congenital toxoplasmosis) *Giardia* spp. (giardiosis) *Cryptosporidium* spp. (cryptosporidiosis)
Counotte et al., 2016	Kyrgyzstan	2013	7 zoonoses	*Echinococcus multilocularis* (alveolar echinococcosis) *Echinococcus granulosus* (cystic echinococcosis) *Toxoplasma gondii* (toxoplasmosis)
van Lier et al., 2016	The Netherlands	2007–2011	32 infectious diseases	*Toxoplasma gondii* (acquired and congenital toxoplasmosis) *Giardia* spp. (giardiosis) *Cryptosporidium* spp. (cryptosporidiosis)

## 3 Economic impact

### 3.1 Methods and metrics

As for health impact, different methods exist for estimating the economic impact of foodborne parasites. The most commonly applied approach measures the cost-of-illness from a societal perspective, taking into account that foodborne parasites have an impact on several stakeholders within the society (Mangen et al., 2015). In cost-of-illness studies, three broad families of cost items are typically defined (Mangen et al., 2010). First, direct healthcare costs defined as the resources provided by the healthcare sector, such as healthcare provider consultations, diagnostic testing, medication, and hospitalization. Second, patient costs (or direct non-healthcare costs) defined as the resources used for healthcare that are not borne by the healthcare system, such as over-the-counter medications and other patient co-payments, and travel expenses to visit a healthcare provider. Third, productivity losses (or indirect non-healthcare costs) defined as the losses due to absenteeism or job loss of patients and their caregivers. A fourth category, the future savings in healthcare costs due to premature death (or indirect healthcare costs), is increasingly being discussed, but not yet routinely included in cost-of-illness studies.

An alternative to cost-of-illness studies are stated preference methods, which elicit general population estimates on the amount people would be willing to pay to prevent (willingness-to-pay; WTP) or be willing to receive to compensate the presence of (willingness-to-accept; WTA) a certain foodborne illness. Estimates are typically derived using discrete choice (or contingent valuation) experiments, in which respondents are asked to choose between two mutually exclusive scenarios, such as the purchase of non-labeled chicken at current market prices, versus the purchase of *Campylobacter*-free chicken at a higher price (Van der Fels-Klerx et al., 2016). Health economists consider stated preferences as the most complete and correct economic welfare measures, as they are not limited to tangible costs but also allow incorporation of changes in consumer welfare associated with pain, distress and inconvenience. Furthermore, they quantify societal preferences, instead of relying solely on technical grounds. Nonetheless, their use has been very limited to date, as the technique is complicated, resource-intensive, and known to suffer from significant between-respondent variability — reflecting differential consumer behavior, which, to some extent, is associated with the respondents' differential ability to pay. Comparability of stated preferences across regions may therefore also be difficult.

On top of the costs linked to the health impact of foodborne parasites (quantified through cost-of-illness or stated preferences), these hazards may also incur an economic impact due to surveillance and other regulatory activities in place to monitor and prevent infection. In the EU, for instance, inspection of pigs at slaughterhouse level for *Trichinella* spp. induces an estimated annual cost of € 25 million (Torgerson, 2013), while the health impact of trichinellosis is negligible (Devleesschauwer et al., 2015b). As many foodborne parasites are zoonotic, livestock losses due to clinical or subclinical infection may further add to the economic burden. In Tanzania, the impact of lower prices for *T*. *solium* infected pigs was estimated at US$ 2.8 million, accounting for 35% of the total economic impact of *T*. *solium* in the country (Trevisan et al., 2017). At a global level, Budke et al. (2006) estimated up to US$ 2 billion livestock production losses due to cystic echinococcosis as a result of liver condemnation, reduction in carcass weight, decrease in hide value, decrease in milk production, and decreased fecundity. Market access (or the lack thereof) may also have significant economic impacts. Furthermore, these knock-on economic effects are not only limited to the population affected by the outbreak. For instance, the first foodborne outbreaks of cyclosporosis in the United States that were associated with raspberries imported from South America resulted in huge economic losses and unemployment in the already marginal economic area of Guatemala where the raspberries originated (Pratdesaba et al., 2001).

Risk ranking based on economic impact may be more tangible and appealing to certain risk managers. It also allows taking multiple dimensions into account, ranging from medical costs, to trade impacts and livestock losses; this however comes at a cost of requiring an even larger amount of data than health impact measures. As for SMPHs, different methodologies and normative values exist for estimating economic impact, leading to limited comparability between studies. Finally, economic impact assessments do not always capture the costs of pain and suffering, yielding cost estimates that are strongly dependent on the economic development level of the study area.

### 3.2 State of the art

Although foodborne parasites are of global concern, there are so far no global risk rankings of foodborne parasites based solely on economic impact. Murrell (1991), Roberts et al. (1994) and Torgerson and Macpherson (2011) aimed at providing a global perspective by reviewing the economic impact of foodborne parasites in multiple countries; however, given the methodological differences between different studies, such reviews do not provide accurate rankings. Furthermore, there are relatively few assessments of the global economic impact of individual foodborne parasites. Budke et al. (2006) estimated global monetary losses resulting from human and livestock cystic echinococcosis. Human-associated direct and indirect costs resulted in a global loss of US$ 764 million, while livestock-associated losses due to liver condemnation and reductions in carcass weight, hide value, milk production, and fecundity resulted in a global loss of US$ 2 billion.

More efforts have been made to conduct risk ranking of foodborne parasites based on economic impact at a national level, in particular in the United States and in the Netherlands. Hoffmann et al. (2012) estimated the annual cost-of-illness in the United States of 14 foodborne pathogens for the year 2009, including that of *T*. *gondii* (US$ 2973 million), *Cryptosporidium parvum* (US$ 47 million), and *C*. *cayetanensis* (US$ 2 million). Jointly, these three foodborne parasites accounted for 21% of the cost-of-illness of all 14 considered pathogens. In a more comprehensive study including 31 foodborne pathogens and a broad category of unspecified agents, Scharff (2012) estimated the cost of foodborne illness in the United States for the year 2010, based on medical costs, monetized QALY losses, and illness-related mortality. The total economic impact was estimated at US$ 78 billion, of which 5% was due to the five included foodborne parasites – i.e., *T*. *gondii* (US$ 3456 million), *Giardia duodenalis* (US$ 282 million), *Cryptosporidium* spp. (US$ 168 million), *C*. *cayetanensis* (US$ 17 million) and *Trichinella* spp. (US$ 2 million). The cost per case was significantly higher for *T*. *gondii* and *Trichinella* spp. (US$ 40,000 and US$ 15,000, respectively), than for the three other included foodborne parasites (< US$ 4000). In the Netherlands, the cost-of-illness of *Cryptosporidium* spp., *Giardia* spp. and *T*. *gondii* was € 8 million, € 11 million, and € 55 million, respectively, accounting for 16% of the economic impact of all considered foodborne pathogens (Mangen et al., 2015). Whereas direct healthcare costs were found to be the dominant component of the cost-of-illness of *T*. *gondii*, productivity losses were the most important component of *Cryptosporidium* spp. and *Giardia* spp. cost-of-illness. This was also noted in the cost estimate for the enormous waterborne outbreak of cryptosporidiosis in Milwaukee in 1993, in which the total cost of outbreak-associated illness was estimated at 96.2 million US dollars, of which 31.7 million US dollars were medical costs and 64.6 million US dollars were productivity losses (Corso et al., 2003).

## 4 Integrating multiple criteria

### 4.1 Methods and metrics

Using a single criterion to rank risks may be insufficient as diseases vary greatly in incidence, clinical manifestations, control measures, transmission potential, and socio-economic impact in animals and humans. *Trichinella* spp., for instance, have a near negligible health impact in Europe, but their economic impact remains important due to continued monitoring and trade implications (Devleesschauwer et al., 2015b). Likewise, *Taenia saginata* mainly poses an economic burden to farmers and society, while the health impact of *T*. *saginata* taeniosis is limited (Laranjo-González et al., 2016). To explore this limitation of single criterion based risk rankings, it may be useful to rank risks according to multiple criteria (Mangen et al., 2010). Mangen et al. (2015), for instance, quantified the burden of foodborne disease in the Netherlands in 2011 using DALYs and cost-of-illness estimates, both at a population and individual level. These different criteria led to four different rankings, with some hazards, most notably *T*. *gondii*, scoring high on multiple rankings.

Several authors have gone beyond the simple comparison of different rankings and proposed methods for combining multiple criteria into a single ranking. The most basic methods rely on the qualitative or semi-quantitative integration of two dimensions, for instance through the construction of a risk matrix – i.e., a two-dimensional combination of two criteria, such as an exposure and a consequence measure, with results ranging from low/low to high/high — but see Cox (2008) for a review of limitations of this method. A specific example of combining two criteria is the translation of DALYs (or QALY losses) into economic impact estimates, and vice versa. Indeed, some authors combined DALYs and economic impact estimates by assuming one DALY to correspond to an economic loss equal to the per capita gross national product (Torgerson et al., 2008). This however implies that the relative value of health loss depends on the wealth of the nation, which may raise equity issues, especially when performing cross-country or global rankings. To address this limitation, Torgerson et al. (2017) introduced the zDALY metric, which is the sum of the DALY metric for human health losses and the equivalent time losses associated with animal losses, defined as the economic impact of the animal losses divided by the per capita gross national product.

More advanced methods make use of the Multi-Criteria Decision Analysis (MCDA) framework. In MCDA, an overall importance measure is constructed based on different criteria, which are assigned weights reflecting their perceived contribution (Cardoen et al., 2009, Havelaar et al., 2010; FAO/WHO (Food and Agriculture Organization of the United Nations and World Health Organization), 2014; Robertson et al., 2015). A typical MCDA exercise thus contains the following steps: identification of pathogens to be ranked; definition of criteria and description of scoring methods (e.g., economic impact, health impact, impact on trade); elicitation of weights for the different criteria (e.g., economic impact may be perceived as less important than health impact, and morbidity or mortality may vary in importance depending on circumstance and perception); and scoring of the criteria, in a quantitative, semi-quantitative, or qualitative way, based on existing data or on expert elicitation. The final ranking is then given by the weighted sum of the different criteria. In addition to this simple additive ranking approach, more advanced MCDA approaches based on utility theory or outranking have been proposed (Geldermann and Schöbel, 2011). Their application to rank foodborne parasites has so far remained limited — but see Ng and Sargeant (2012) for an application of conjoint analysis and Kadohira et al. (2015) for an application of the analytic hierarchy process method, both examples of utility based methods. Despite potential subjective elements, MCDA provides a reproducible, standardized and transparent framework for ranking risks, and is consequently used in multiple sectors (Cardoen et al., 2009, Behzadian et al., 2010, Anderson et al., 2011) — the European Centre for Disease Prevention and Control, for instance, prepared an MCDA toolkit for prioritizing infectious disease threats (ECDC (European Centre for Disease Prevention and Control), 2017). MCDA also allows the simultaneous consideration of criteria that are quantitative (e.g., incidence, case-fatality ratio) with others for which only semi-quantitative measures are available (e.g., trade relevance scored as “no relevance”, “some relevance”, and “high relevance”) (Ruzante et al., 2010; FAO/WHO (Food and Agriculture Organization of the United Nations and World Health Organization), 2014). MCDA as such does not strive for an absolute numerical reflection of the actual situation; rather, it strives to compare the relative importance of foodborne parasites using a comprehensive consideration of all components that are deemed relevant for that importance.

### 4.2 State of the art

**Fig. 3:**
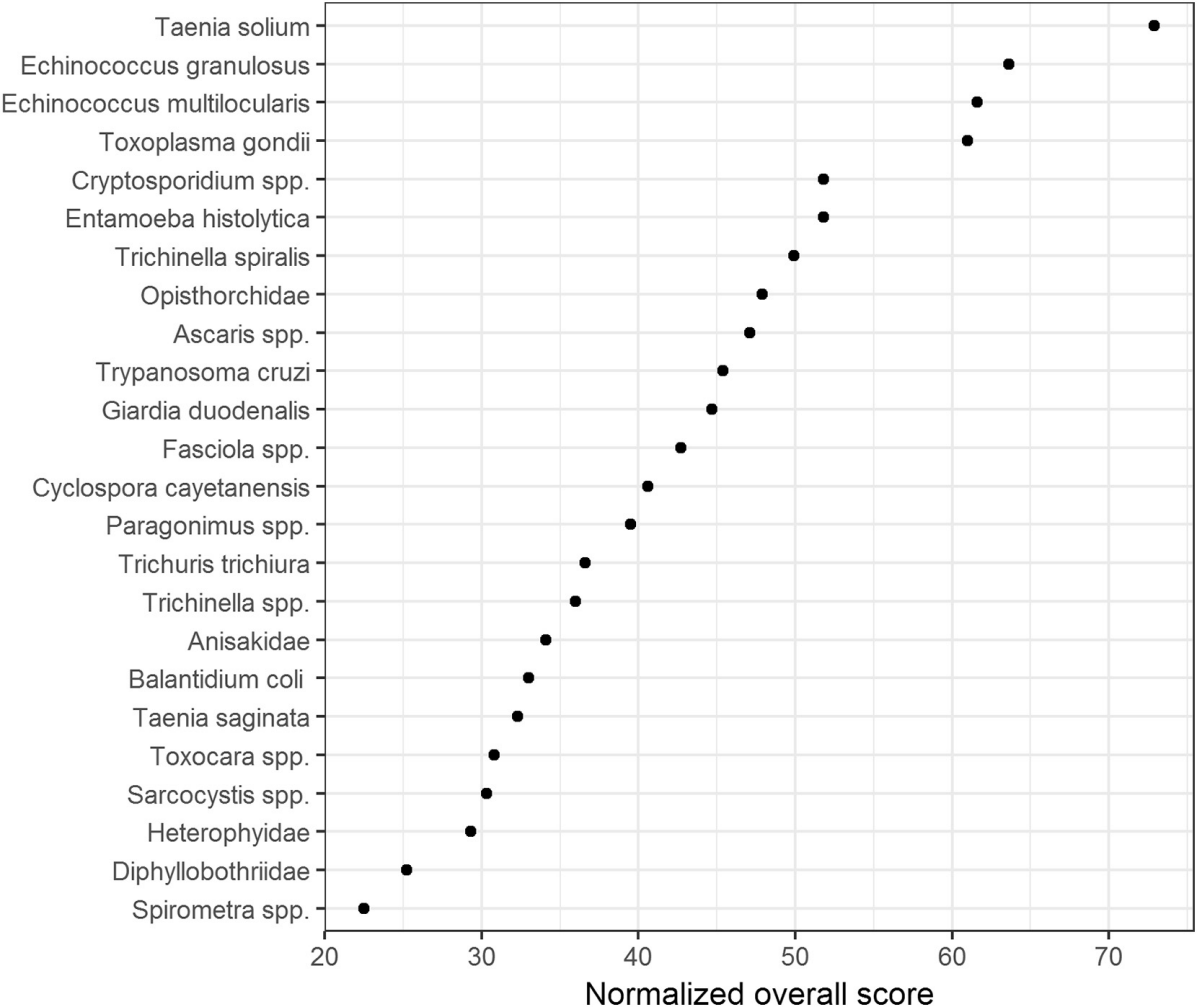
Global ranking of foodborne parasites using multi-criteria decision analysis (FAO/WHO (Food and Agriculture Organization of the United Nations and World Health Organization), 2014). The normalized overall scores are weighted sums of normalized criteria scores and weights elicited from expert meeting participants.

In 2012, the Food and Agriculture Organization of the United Nations (FAO) and World Health Organization (WHO) jointly organized a multicriteria-based ranking of foodborne parasites at a global level. The exercise considered 24 foodborne parasites, narrowed down from an initial list of 93, which were scored according to seven criteria, including criteria related to health impact, trade relevance, and impacts on economically vulnerable communities. Fig. 3 presents the obtained global ranking of foodborne parasites, confirming the importance of *T*. *solium* at a global level, closely followed by *E*. *granulosus*, *E*. *multilocularis*, and *T*. *gondii*. Recently, the FAO/WHO MCDA exercise has been repeated at a regional level. Robertson et al. (2015) used the MCDA approach to perform a risk ranking of foodborne parasites in India, while Bouwknegt et al. (2017) used the approach to generate European risk rankings. As expected, the results differed from those of the global ranking, as the epidemiology and impact of foodborne parasites is known to vary considerably between countries and regions, while perhaps also the perception of different criteria may vary across cultures. Specifically, in India *T*. *solium* was ranked highest, followed by *Cryptosporidium* spp. and *E*. *granulosus*, while in Europe, *E*. *multilocularis* ranked first, followed by *T*. *gondii* and *Trichinella spiralis*. The criteria weights obtained in the global and European exercises were relatively similar, while the Indian exercise elicited a lower weight for morbidity severity but a higher weight for the number of illnesses (Table 4).

**Table 4:** Criterion weights obtained in three multi-criteria decision analyses of foodborne parasites.

Scoring criterion	Global^a^	India^b^	Europe^c^
Number of global food-borne illnesses	0.22	0.30	0.23
Global distribution	0.14	0.13	0.13
Morbidity severity	0.22	0.14	0.23
Case-fatality ratio	0.15	0.15	0.15
Increasing illness potential	0.07	0.12	0.10
Trade relevance	0.10	0.07	0.09
Impacts on economically vulnerable communities	0.10	0.08	0.07

a FAO/WHO (Food and Agriculture Organization of the United Nations and World Health Organization) (2014).

b Robertson et al. (2015).

c Bouwknegt et al. (2017).

Several other MCDA exercises have been conducted to rank foodborne parasites at national or regional level (Table 5). These studies had a broad scope, focusing for instance on zoonotic diseases or, even more broadly, on communicable diseases. Foodborne parasites generally scored relatively low, although it should be noted that the resulting rankings are not necessarily comparable, given the differences in pathogens to rank, methodologies, and criteria. Inclusion of different pathogens to rank can for instance change the relative ranking of the other pathogens that are the same. Likewise, different criteria will result in different values being expressed.

**Table 5:** National and regional risk ranking of foodborne parasites and other pathogens based on multi-criteria decision analysis.

Reference	Location	Scope	Criteria	Ranking of foodborne parasites
Cardoen et al., 2009	Belgium	51 zoonotic pathogens	1. Severity of the disease for humans. 2. Occurrence of the disease in the Belgian population. 3. Occurrence in live animals in Belgium. 4. Severity of the disease for animals and commercial and economic impact of disease for the sector. 5. Occurrence of the agent in food or in carcasses.	• High importance: none. • Significant importance: *Toxoplasma gondii*, *Cryptosporidium parvum*, *Echinococcus granulosus*, *Echinococcus multilocularis*, *Fasciola hepatica*, *Giardia duodenalis.* • Moderate importance: *Taenia saginata*, *Trichinella* spp., *Sarcocystis bovihominis.* • Low importance: *Anisakis simplex*, *Sarcocystis suihominis*, *Taenia* spp. (other than saginata), *Dioctophyma renale*, *Clonorchis sinensis*, *Balantidium coli*, *Diphyllobothrium*, *Linguatula serrata*.
Havelaar et al., 2010	The Netherlands	86 zoonotic pathogens	1. Probability of introduction into the Netherlands. 2. Transmission in animal reservoirs. 3. Economic damage in animal reservoirs. 4. Animal-human transmission. 5. Transmission between humans. 6. Morbidity (disability weight). 7. Mortality (case-fatality ratio).	• High priority: *Toxoplasma gondii.* • Medium priority: *Echinococcus multilocularis*, *Trichinella* spp. • Low priority: *Cryptosporidium parvum*, *Ascaris suum*, *Giardia duodenalis*, *Toxocara canis*/*cati*, *Taenia solium*, *Echinococcus granulosus*, *Anisakis simplex*, *Taenia saginata*, *Fasciola hepatica.*
Balabanova et al., 2011	Germany	127 pathogens	1. Incidence (including illness and symptomatic infection). 2. Work and school absenteeism. 3. Health care utilization (primary care and hospitalization). 4. Chronicity of illness or sequelae. 5. Case fatality rate. 6. Proportion of events requiring public health actions. 7. Trend. 8. Public attention (including political agenda and public perception). 9. Prevention possibilities and needs (including vaccines). 10. Treatment possibilities and needs (including AMR).	• Highest priority: none. • High priority: *Cryptosporidium parvum*/*hominis*, *Giardia duodenalis*, *Toxoplasma gondii.* • Medium priority: *Trichinella spiralis.* • Low priority: *Cyclospora cayetanensis*, *Entamoeba histolytica*, Helminhts (flukes), Helminths (nematodes), Helminths (tapeworms).
Ng and Sargeant, 2012	Canada and US	62 zoonoses	1. Case-fatality in humans. 2. Incidence of the disease in the Canadian/US human population in the last five years. 3. Case-fatality in animals. 4. Incidence of the disease in the Canadian/US animal population in the last five years. 5. Severity of illness in humans. 6. Disease trend in Canada/US in the last five years in humans. 7. Transmission potential between humans. 8. Duration of illness in humans. 9. Transmission potential from animals to humans. 10. Disease trend in Canada/US in the last five years in animals. 11. Economic burden in humans. 12. Transmission potential from humans to animals. 13. Duration of illness in animals. 14. Transmission potential between animals. 15. Economic and social burden on trade in animals. 16. Severity of illness in animals. 17. High risk groups in humans. 18. Control measures in humans. 19. Control measures in animals. 20. How much is known scientifically about the disease. 21. High risk groups in animals.	• Canada: Cryptosporidiosis (14), Giardiosis (19), Toxoplasmosis (22), Echinococcosis (37), Toxocarosis (38), Trichinellosis (49), Cystiocercosis/Taeniosis (57), Cyclosporosis (59). • US: Cryptosporidiosis (23), Toxoplasmosis (32), Giardiosis (33), Echinococcosis (41), Toxocarosis (43), Cysticercosis/Taeniosis (45), Trichinellosis (55), Cyclosporosis (57).
Humblet et al., 2012	Europe	100 animal diseases and zoonoses	57 criteria, including 17 for epidemiology, 8 for prevention/control, 16 for economy/trade, 12 for public health, and 4 for society	• High importance: Echinococcosis/hydatidosis. • Significant importance: Porcine cysticercosis, Trichinellosis. • Moderate importance: none. • Relative low importance: none.
Dahl et al., 2015	Sweden	106 pathogens	1. Incidence (including illness, symptomatic infections, asymptomatic infections but not carriership or normal flora). 2. Work and school absenteeism. 3. Health care utilization (primary health care and hosptitalization). 4. Chronicity of illness or sequelae. 5. Case fatality rate. 6. Proportion of events requiring public health actions. 7. Trend. 8. Public attention (including political agenda and public perception). 9. Prevention possibilities and needs (including vaccines). 10. Treatment possibilities and needs (including AMR).	• Highest priority: *Echinococcus multilocularis.* • High priority: *Cryptosporidium parvum*/*hominis*, *Giardia duodenalis.* • Medium priority: *Echinococcus granulosus*, *Enterobius vermicularis*, *Toxoplasma gondii*, *Trichinella spiralis.* • Low priority: *Entamoeba histolytica*, Helminhts (tapeworms), Helminths (flukes), Helminths (nematodes).
Kadohira et al., 2015	Japan	98 zoonoses	1. No. of human cases/year (incidence). 2. Human-to-human spread. 3. Case fatality rate. 4. Availability of diagnostic test. 5. Treatment. 6. Preventive methods. 7. Frequency of entry to Japan.	Echinococcosis: 16/20 most important zoonoses

## 5 General considerations

To conclude, we want to present three general considerations when designing a risk ranking exercise of foodborne parasites.

• **Whose ranking perspective**? The aim of risk ranking exercises is to prioritize for decision making certain hazards, hazard-commodity pairs, or exposure routes for a given hazard, based on their perceived importance. As different stakeholders have their own prioritization objectives, the outcome of such exercises is necessarily context-dependent. Consequently, there is no unique or intrinsically correct ranking of risks. Furthermore, when designing a risk ranking exercise, it is important that the precise question and goal being addressed are defined explicitly, and from which perspective, along with the intended group who will use the ranking and act upon it. When opting for MCDA, quantifying the criteria weights should be conducted along this line by considering whose perception on relative importance among criteria needs to be reflected in the ranking.
• **Which risk metric**? As outlined in the review, there are different metrics, and methods, that can be used to define and rank risks. Different metrics have different philosophical implications, and result in different rankings. It is thus important to give considerable thought to the definition of metrics, and to ensure that the chosen metric and method fulfill the needs of the target audience. When multiple criteria are of interest in defining a ranking, methods relying on SMPHs or MCDA provide the most appropriate quantifications.
• **What to rank**? Obviously, the prior definition of included foodborne parasites will have an impact on the final ranking. Furthermore, specific foodborne parasites may have multiple appearances, such as congenital versus acquired toxoplasmosis; whether or not to consider these as one or multiple entities is an important prior choice. A further major consideration is whether or not the included food-related parasites should be ranked solely according to their foodborne transmission; and if so, whether drinking water is considered in the definition of food.

## 6 Conclusion

Risk ranking of foodborne parasites is increasingly performed to aid priority setting at global, regional, and national levels. Different risk ranking metrics and methods are available, ranging from single measures of health or economic impact, to complicated, but more complete, multi-criteria assessments. When designing a risk ranking exercise of foodborne parasites, it is important to consider the target audience and the reason for which the ranking is done, the choice of metrics and methods, and the prior definition of what to rank.
